# Tissue Plasminogen Activator for preclinical stroke research: Neither “rat” nor “human” dose mimics clinical recanalization in a carotid occlusion model

**DOI:** 10.1038/srep16026

**Published:** 2015-11-02

**Authors:** Amelia J. Tomkins, Rebecca J. Hood, Christopher R. Levi, Neil J. Spratt

**Affiliations:** 1 School of Biomedical Sciences & Pharmacy, Medical Sciences Building, University of Newcastle, University Drive, Callaghan, NSW, 2308, Australia; 2 Hunter Medical Research Institute, Lot 1 Kookaburra Circuit, New Lambton Heights, NSW, 2305, Australia; 3 Hunter New England Local Health District, Department of Neurology, Lookout Road, New Lambton Heights, NSW, 2305, Australia; 4 School of Medicine and Public Health, University of Newcastle, Newcastle, NSW, Australia

## Abstract

Tissue plasminogen activator (tPA) is the only approved thrombolytic therapy for acute ischemic stroke, yet many patients do not recanalize. Enhancing thrombolytic efficacy of tPA is a major focus of stroke research. Traditionally, a “rat dose” of 10 mg/kg has been used in rodent models. Recent studies suggested that the clinical “human” dose (0.9 mg/kg) may better mimic clinical recanalization. These studies only compared the rat and clinical doses, and so we aimed to test recanalization efficacy of multiple tPA doses ranging from 0.9 to 10 mg/kg in a model of endothelial injury and vessel stenosis. The common carotid artery of rats was crushed and stenosed to allow *in-situ* occlusive thrombus formation (Folt’s model of ‘physiological’ thrombus). Intravenous tPA was administered 60 minutes post-occlusion (n = 6-7/group). Sustained recanalization rates were 0%, 17%, 67% and 71%, for 0.9, 1.8, 4.5, and 10 mg/kg, respectively. Median time to sustained recanalization onset decreased with increasing dosage. We conclude that 10 mg/kg of tPA is too effective, whereas 0.9 mg/kg is ineffective for lysis of occlusive thrombi formed *in situ*. Neither dose mimics clinical tPA responses. A dose of 2x the clinical dose is a more appropriate mimic of clinical tPA recanalization in this model.

Tissue plasminogen activator (tPA) is the only approved thrombolytic therapy for acute ischemic stroke. Early recanalization of occluded vessels is associated with improved clinical outcome, yet less than 50% of all stroke patients treated intravenously with tPA will successfully recanalize^1^. In the setting of carotid artery occlusion, tPA is even less effective, with recanalization rates of 10–30%^2^,^3^. There is a great need for improved therapies for stroke and one approach has been to enhance thrombolysis with adjuvant therapies such as sonothrombolysis^4^. For any new or adjuvant thrombolytic therapy, rigorous preclinical testing should occur and aim to mimic the clinical conditions of ischemic stroke and tPA efficacy. There has been controversy regarding what a “human equivalent” dose of tPA is for preclinical research—particularly in rodents, in which most such studies are performed.

Traditionally, a dose of 10 mg/kg tPA has been used for rodents. This “rat dose” was based on an *in vitro* study from the early 1980’s that demonstrated that the fibrinolytic system of rats is 10-fold less sensitive than humans^5^. Two recent comparisons of rat and clinical doses in rats^6^ and mice^7^ both indicated that the clinical dose was a better mimic of the clinical situation^6^,^7^. Additional doses were not compared in these studies. The typical method for determining a “human equivalent” dose of any therapeutic utilizes a conversion based on body surface area of humans to the target species^8^. For rats, this conversion requires multiplication of the human dose (0.9 mg/kg) by 6.2, indicating a “human equivalent” dose of 5.58 mg/kg tPA for rats. However, this conversion does not take in to account additional factors that may affect the fibrinolytic process other than body size, and is not generally used when converting doses of tPA for stroke research.

Multiple methods of forming experimental thrombi exist for preclinical stroke models. Formation of thrombi *in situ* or *ex vivo* and the presence or absence of added pro-thrombotic factors, such as thrombin and/or CaCl_2_, leads to variability of final thrombus composition. All of these factors play a key role in the overall thrombolytic susceptibility of the thrombus and therefore the variability of tPA efficacy between studies^9^,^10^. Ideally, a preclinical model for testing stroke thrombolytics and thrombolytic enhancers should use thrombi that closely mimic human stroke thrombi and have similar recanalization rates.

For this study, we developed a method of physiological thrombus formation by endothelial injury and stenosis of the carotid artery in rats. To determine which tPA dose best reflects clinical recanalization rates in this model, we aimed to investigate sustained recanalization rates of varying doses of tPA ranging from the clinical dose to the traditional rat dose. Time to sustained recanalization was a secondary outcome.

## Results

**Figure 1:**
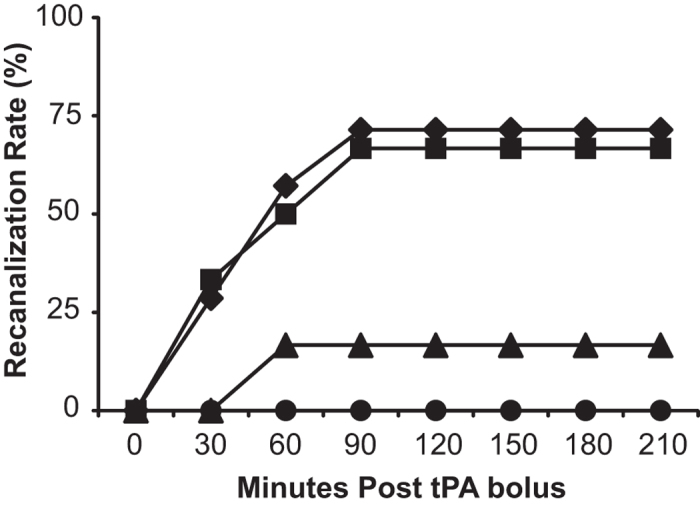
Recanalization rates at varying doses of tPA. Rats with carotid artery occlusion were administered intravenous tPA at the clinical dose (0.9 mg/kg, ●), 2x the clinical dose (1.8 mg/kg, ▲), 5x the clinical dose (4.5 mg/kg, ■), and the rat dose (10 mg/kg, ♦). Treatment began 60 minutes post-occlusion. n = 6 per group for 0.9, 1.8 and 4.5 mg/kg, n = 7 for 10 mg/kg. Data presented as the percentage of animals with sustained recanalization per group. Fisher’s exact test, p = 0.015.

To test the thrombolytic efficacy of varied doses of tPA on a physiological thrombus we used a rat model of carotid occlusion with a mild underlying stenosis. Recanalization was monitored every 30 minutes post-tPA delivery to 4.5 hours post-occlusion. Sustained recanalization, defined as recanalization without reocclusion, was observed in 0% of 0.9 mg/kg treated rats (0/6), 17% of 1.8 mg/kg treated rats (1/6), 67% of 4.5 mg/kg treated rats (4/6), and 71% of 10 mg/kg treated rats (5/7) (Fisher’s exact test, p = 0.015, Fig. 1). Recanalization/reocclusion was observed in 2 animals, both in the 10 mg/kg dosage group. In pilot experiments we found that recanalization was easily confirmed (as in Fig. 3B). However accurate quantification of the degree of recanalization was not possible because coupling of the flow probe to the vessel with saline caused fluctuations in the baseline of the flow trace (data not shown). We did not see evidence of major changes in flow once vessels did recanalize. Therefore, in the interests of accuracy, we chose sustained recanalization as the marker of recanalization success based on dose, rather than attempting to quantify percentage recanalization.

**Figure 2:**
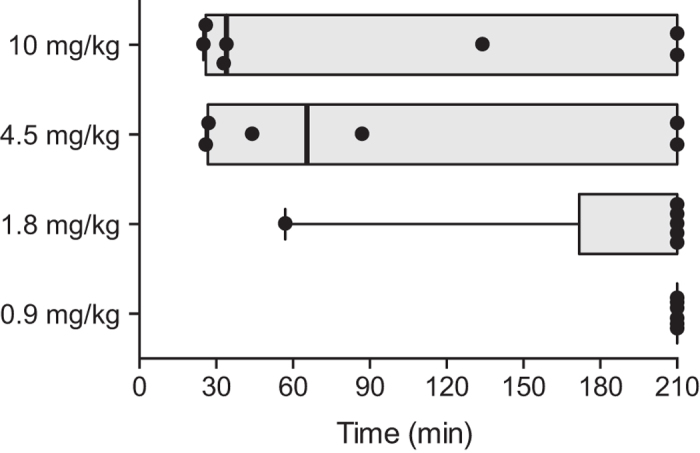
Time to sustained recanalization. Sustained recanalization was defined as flow return without reocclusion to experiment end (4.5 hours post-occlusion). Individual animal times are presented as dots. Any animal still occluded at the end of the experiment was designated the maximal time to recanalization (210 min). Data presented as median (solid band) and interquartile range (grey box). There was a significant overall difference of dose, log rank test, p = 0.017.

Median times to recanalization (interquartile range) from start of tPA treatment were 210 (210–210) minutes for 0.9 mg/kg treated rats, 210 (210–210) minutes for 1.8 mg/kg treated rats, 65.5 (26–210) for 4.5 mg/kg treated rats, and 34 (27–210) minutes for 10 mg/kg treated rats (Log Rank Test, p = 0.017, Fig. 2). Earliest recanalization onset was 25 minutes (10 mg/kg), with no recanalization occurring beyond 87 minutes (27 minutes after tPA treatment end) in any group.

No animals were excluded. One animal died just prior to the final observation (10 mg/kg group). This animal had fluctuating body temperatures throughout surgery and high temperatures (>39 °C) leading up to its death, with no other explanation found. It was included in the primary analysis. Re-analysis excluding this animal gave a recanalization rate of 83% (instead of 71%) for this group, but made no material difference to the primary findings.

## Discussion

In this study, we found that the traditional rat dose of tPA (10 mg/kg) was highly effective for carotid artery recanalization (71% recanalization rate). This is far superior to what is achievable in clinical stroke, where recanalization rates are <50% for middle cerebral artery (MCA) occlusion^1^ and only 10–30% for occlusions of the carotid arteries^2^,^3^. The clinical dose of tPA (0.9 mg/kg) caused no recanalization in our model, which is also not reflective of the clinical situation. We found a 2x clinical dose (1.8 mg/kg) to better reflect clinical recanalization rates. We observed a possible ceiling effect at doses at and above 4.5 mg/kg (5x clinical dose). Our findings are in keeping with previous work showing that the rat fibrinolytic system is less sensitive than the human system. However, they suggest that the 10-fold difference in sensitivity found *in vitro*^5^ may overestimate the *in vivo* situation.

A “human equivalent” dose of tPA for preclinical research that results in recanalization rates reflective of the clinical setting has not previously been determined. We chose a range of doses spanning across those used in previous studies, with multiples of the clinical dose (i.e. 2x and 5x the clinical dose). Although the thrombolytic efficacy of tPA has been well established clinically, its efficacy is subobtimal^1^ and the growing field of research studying thrombolytic enhancers requires a preclinical dose of tPA that reflects the clinical response rates. Recent studies comparing clinical to rat dose reported the clinical dose to be a better clinical mimic, but these studies did not investigate additional doses^6^,^7^. The high recanalization rates we saw with the 10 mg/kg “rat” dose are similar to rates reported in other studies using this dose (67–100% in various stroke models^7^,^11^,^12^,^13^). These rates are not reflective of clinical recanalization and are particularly unsuited to studies of thrombolytic enhancer therapies, since there is little scope for additional benefit. Additionally, we found a possible ceiling effect of tPA dose at 4.5 mg/kg, with no significant increase in recanalization rates at the higher dose. Such an effect has also previously been reported for doses above 10 mg/kg^14^. For a “human equivalent” tPA dose, our study indicates that a 2x clinical dose (1.8 mg/kg) in this model with 17% recanalization rates best reflects clinical recanalization of carotid artery occlusion for which clinical rates are between 10–30%^2^,^3^. To achieve the higher end of this clinical range, a dose between 1.8 mg/kg and 4.5 mg/kg may be necessary. However, this highlights that the previously accepted doses are not ideal to mimic clinical rates. In other situations, such as MCA occlusion, clinical recanalization rates are higher than carotid occlusion^1^. Differing clot compositions and co-morbidities also affect recanalization efficacy with tPA^15^,^16^. It is likely that tPA doses required to mimic clinical recanalization rates will differ with regards to the model choice, incorporating clot type, co-morbidities, and species. We recommend that researchers aiming to find improved thrombolytic or recanalization therapies over tPA alone, need to determine the “human equivalent” dose for use in their chosen model that generates recanalization rates that correlate with the clinical conditions being tested.

We chose a model of naturally forming thrombi in order to create as ‘physiological’ a model as possible. This clot composition is the likely explanation for the complete lack of recanalization we saw with clinical dose tPA, in contrast to previous studies. Such studies have tended to use spontaneously formed thrombi, or other clot types with high sensitivities to tPA thrombolysis. Thrombus composition is well known to affect the efficacy of tPA lysis^9^,^10^. Our model, based on the Folt’s method, produces physiological thrombi that are platelet rich^17^. Platelet rich clots are histologically better mimics of clinical thrombi^18^ and are more resistant to thrombolysis than many other experimental thrombi^19^,^20^,^21^.

Recanalization was chosen as the primary outcome for this study because it is the major effect by which tPA causes clinical improvement^1^. In rat models of stroke, recanalization is not always reported due to difficulties in directly monitoring recanalization intracranially in the rat. The extracranial nature of our chosen model allowed direct real time monitoring of recanalization. We observed earlier onset time to recanalization with increasing doses of tPA. With early recanalization being predictive of good clinical outcome^1^, it is beneficial for enhancer therapies to not only increase the rates of recanalization, but to reduce the time to sustained recanalization onset. At higher doses, recanalization onset is already early (median time 34 minutes for 10 mg/kg), leaving little room for improvement if testing alternate therapies. Functional outcome was intentionally not included in this study because there is already convincing evidence that tPA produces good functional outcome in rodents^1^,^22^. We know from clinical studies that functional improvement is highly correlated with recanalization of the occluded artery. The purpose of this study was to determine whether the previously accepted “rat dose” causes recanalization rates too high to provide any scope for improvement when testing thrombolytic enhancers.

In conclusion, we have found that both rat and clinical doses of tPA are not reflective of clinical recanalization rates in a model of naturally forming thrombus. The rat dose was above that producing a “maximal” effect of recanalization. The no response observed with clinical tPA dose confirms that the rat fibrinolytic system is less sensitive to humans, but not to the 10-fold degree previously accepted. Neither dose appeared ideal for testing thrombolytic enhancers. For this model of carotid occlusion in rats, we propose a 2x clinical dose (1.8 mg/kg) to be best reflective of clinical recanalization rates.

## Methods

### Animals

This study was approved by the Animal Care and Ethics Committee of the University of Newcastle, Australia (Approval No. A-2010-128) and performed in compliance with requirements of the Australian Code of Practice for the Care and Use of Animals for Scientific Purposes^23^. Male outbred Wistar rats (n = 25; 338–433 g; Animal Resources Centre, Perth, Australia) were anesthetised with 5% Isoflurane, and maintained with 1.5–2% in 30/70% O_2_/N_2_ through a nose cone and rectal temperature was maintained at 37 °C with a feedback controlled heat mat (Faculty of Health Workshop, University of Newcastle, Australia). Heart rate, respiration and oxygen saturation were monitored throughout surgery.

### Carotid Artery Occlusion

**Figure 3:**
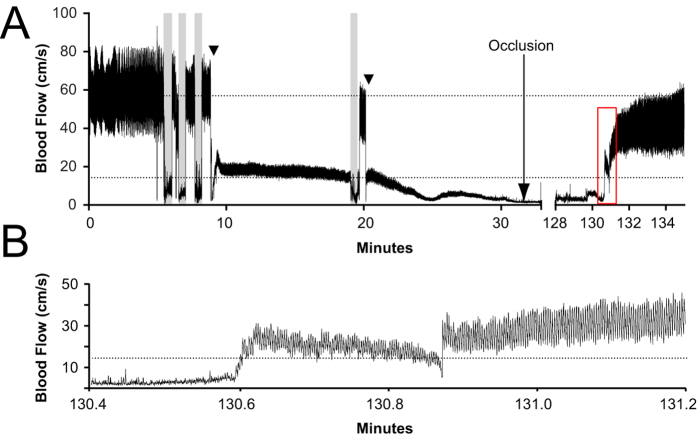
Doppler flow of crush and occlusion with recanalization. Representative example of Doppler flow indicating crush injury and stenosis followed by occlusion (**A**). Grey shaded bars indicate 3 × 30 second crush cycles, including a recrush after 10 minutes of continuous flow. Triangles (▼) indicate tightening of silk suture to create stenosis and achieve 75% flow reductions. Dashed horizontal lines indicate baseline flow of 57 cm/s (**A**) and 25% baseline flow of 14.25 cm/s (**A**,**B**). Recanalization was observed at 130.6 minutes (**A**,**B**). The red box in (**A**) indicates the area zoomed in for (**B**) to show clear change from occluded “noise” to flow trace, representing recanalization. Recanalization was also confirmed by the return of audible Doppler flow correlating with flow trace.

To create a physiological thrombus to occlude the carotid artery, a modification of the Folt’s model^17^ was used with a mild underlying stenosis. The right carotid arteries were exposed and the internal carotid artery ligated to avoid risk of thromboembolism to the brain. Resultant strokes from carotid thromboembolism are highly variable, as in stroke patients, and could potentially confound experiments. A 20-MHz, 0.8 mm Doppler flow probe (Iowa Doppler Products, USA) was positioned over the external carotid artery to monitor blood flow. Baseline flow was recorded for 5 minutes prior to injury using LabChart 7 software (ADInstruments, Australia). The common carotid artery was crushed three times (30 seconds with 30 second rest intervals, Fig. 3A) using haemostats guarded with tape to disrupt the endothelium, exposing pro-thrombotic factors. A double looped 5-0 silk suture was placed over the site of injury, and tightened to create stenosis following final crush. This stenosis reduced blood flow by 75% of pre-crush baseline flow. Flow was monitored for cyclic flow patterns until complete occlusion was achieved (Fig. 3A). An additional 30 second crush was made over the stenosis if: 1) continuous flow was maintained for 10 minutes after final crush, or 2) if cyclic flow patterns were observed for 30 minutes post-crush with no complete occlusion. No more than two additional crushes were performed in any animal. The tie was loosened 45 minutes after stable occlusion to establish a mild stenosis. Flow spontaneously returned pre-treatment in two animals. In one, the vessel was re-crushed, while the other reoccluded spontaneously within minutes. Sixty minute stable occlusion was again monitored before treatment.

### Treatment Groups

At 60 minutes post-occlusion, animals were intravenously administered tPA (Alteplase, Boehringer Ingelheim, Australia) at the clinical dose (0.9 mg/kg, n = 6), 2x the clinical dose (1.8 mg/kg, n = 6), 5x the clinical dose (4.5 mg/kg, n = 6), or the rat dose (10 mg/kg, n = 7) as a 10% bolus over 1 minute, with the remainder infused over 1 hour.

The 0.9 mg/kg and 10 mg/kg groups were part of a separate study in which they had been randomised to either tPA or tPA+ultrasound treatment (tPA + ultrasound groups not presented here). The additional animals were randomized to either 1.8 or 4.5 mg/kg tPA for this study.

### Recanalization

The primary outcome for this study was recanalization rates for each group. Recanalization was monitored every 30 minutes after tPA onset until 4.5 hours post-occlusion and presented as the total number of animals with sustained recanalization at experiment end per group. The end point of 4.5 hours was chosen based on the clinical time window for tPA treatment inclusion. Recanalization was reported as sustained when recanalization occurred and flow remained until the final observation time. Recanalization/reocclusion was reported when flow was observed at one time point and no flow observed at the next.

A secondary outcome of time to sustained recanalization was determined retrospectively from Labchart files by a blinded observer and was reported as the time that the flow trace returned to normal waveform post tPA treatment start, with the flow trace continuing as normal to experiment end (Fig. 3B).

### Statistics

Sustained recanalization rates at endpoint were analysed for statistical significance using Fisher’s exact test. Time to sustained recanalization onset was analysed by survival analysis and log-rank test. Statistical significance was considered to be a p-value < 0.05.

## Additional Information

**How to cite this article**: Tomkins, A. J. *et al.* Tissue Plasminogen Activator for preclinical stroke research: Neither “rat” nor “human” dose mimics clinical recanalization in a carotid occlusion model. *Sci. Rep.* **5**, 16026; doi: 10.1038/srep16026 (2015).
